# Clinical Profile and Demographic Distribution of Synchysis Scintillans: An Electronic Medical Record-Driven Big Data Analytics From an Eye Care Network in India

**DOI:** 10.7759/cureus.25982

**Published:** 2022-06-16

**Authors:** Anthony Vipin Das, Yogita Kadam, Mudit Tyagi

**Affiliations:** 1 Department of eyeSmart EMR & AEye, L V Prasad Eye Institute, Hyderabad, IND; 2 Srimathi Kanuri Santhamma Centre for Vitreoretinal Diseases, L V Prasad Eye Institute, Hyderabad, IND

**Keywords:** india, big data, electronic medical records, vitreous, synchysis scintillans

## Abstract

Introduction: To describe the demographics and clinical profile of synchysis scintillans in patients presenting to a multitier ophthalmology hospital network in India.

Methods: This cross-sectional hospital-based study included 3,082,727 new patients presenting between August 2010 and December 2021. Patients with a clinical diagnosis of synchysis scintillans in at least one eye were included as cases. The data were collected using an electronic medical record system.

Results: Overall, 93 (0.003%) patients were diagnosed with synchysis scintillans. About half of the patients were male (50.54%) and had unilateral (81.72%) affliction. The most common age group at presentation was during the seventh decade of life with 38 (40.86%) patients. The overall prevalence was higher in patients from a higher socioeconomic status (0.003%) presenting from the metropolitan geography (0.005%) and in retired individuals (0.018%). A systemic history of diabetes mellitus was documented in 13 (13.98%) patients and hypertension was documented in 15 (16.13%) patients. The majority of the eyes had mild or no visual impairment (<20/70) in 74 (67.27%) eyes. The most commonly associated ocular comorbidity was cataract in 61 (55.45%) eyes followed by glaucoma in eight (7.27%) eyes. Among the surgical interventions performed for the ocular comorbidities, cataract surgery was performed in nine (8.18%) eyes, and vitreoretinal surgery and trabeculectomy were performed in two (1.82%) eyes each.

Conclusion: Synchysis scintillans equally affect males and females presenting during the seventh decade of life and is predominantly unilateral. The majority of the eyes have mild or no visual impairment and over half of the eyes have an associated cataract.

## Introduction

Synchysis scintillans is a degenerative process that is characterized by the deposition of cholesterol crystals in the vitreous cavity, subretinal space, and rarely in the anterior chamber. It is also called cholesterosis bulbi since these crystals are composed of cholesterol [1]. It is characterized by many flat crystalline bodies suspended in a degenerative vitreous fluid. Synchysis scintillans can be secondary to eye trauma, long-term cataract, recurrent intraocular inflammation, hemorrhage or hyphema, secondary glaucoma, or vascular disorders, and can be found both in the anterior and posterior chamber of the eye [2,3]. It can be unilateral or bilateral and is usually seen in the third decade [4]. The vitreous is accompanied by many physiological changes due to aging. The tiny yellowish white floating crystals of cholesterol in the liquified vitreous aid in settling the crystals in a dependent position. There is a redox imbalance seen with increased oxidative modifications and loss of hyaluronic acid in the vitreous that might be age related [5]. There are very few reports in the literature that aid in a comprehensive understanding of this rare disease characterized by a “Golden Shower” as they are limited to a few case reports. There is a paucity of literature on the prevalence and demographic distribution of synchysis scintillans in the Indian population. The purpose of the study is to present the clinical and demographic profile of synchysis scintillans at a large multitier ophthalmology network in India using electronic medical record-driven analytics.

## Materials and methods

Study design, period, location, and approval

This cross-sectional observational hospital-based study included all patients presenting between August 2010 and December 2021 to a multitier ophthalmology network located in India [6]. The patient or the parents or guardians of the patient filled out a standard consent form for electronic data privacy at the time of registration. None of the identifiable parameters of the patient were used for the analysis of the data. The clinical data of each patient who underwent a comprehensive ophthalmic examination was entered into a browser-based electronic medical records system (eyeSmart EMR) by uniformly trained ophthalmic personnel and supervised by an ophthalmologist using a standardized template [7]. The study adhered to the Declaration of Helsinki and was approved by the Institutional Ethics Committee.

Cases

A total of 3,082,727 new patients presented to the secondary and tertiary centers of the multitier ophthalmology network during the study period. The eyeSmart EMR was screened for patients with a documented ocular diagnosis of synchysis scintillans in one or both eyes. A total of 93 patient records were identified using this search strategy and were labeled as cases. A total of 110 eyes diagnosed with synchysis scintillans in the above patients were further analyzed for clinical information.

Data retrieval and processing

The data of 93 patients included in this study were retrieved from the electronic medical record database and segregated into an excel sheet. The columns included the data on patient demographics, clinical presentation, ocular diagnosis, and treatment information and were exported for analysis. The excel sheet with the required data was then used for analysis using the appropriate statistical software. Standardized definitions were used for occupation and socioeconomic status [8]. Visual acuity was classified according to the WHO guidelines [9].

Statistical analysis

Descriptive statistics using mean ± standard deviation and median with interquartile range (IQR) were used to elucidate the demographic data. All tables for age, gender, visual acuity, and clinical features were drawn using Microsoft Excel (Microsoft Corporation 2018. Redmond, USA). Chi-square test (StataCorp. 2015. Stata Statistical Software: Release 14. College Station, TX: StataCorp LP) was used for univariate analysis to detect significant differences in the distribution of demographic features between patients with synchysis scintillans and the overall population.

## Results

Prevalence

Of the 3,082,727 new patients who presented across the eye care network during the study period, 93 patients were diagnosed with synchysis scintillans in at least one eye, translating into a prevalence rate of 0.003% (95% CI: ±0.00003%) or 30/million population.

Age

**Figure 1:**
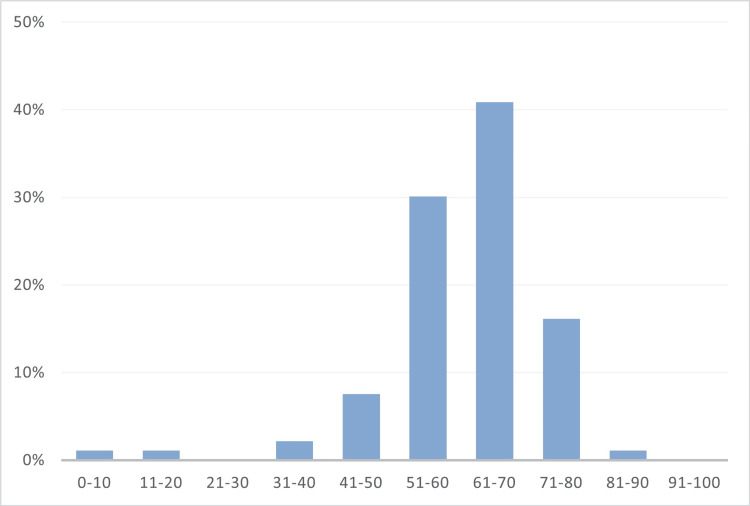
Decade-wise distribution of patients with synchysis scintillans

The mean age of the patients was 60.64±11.85 years while the median age was 62 (IQR: 55-68) years. The most common age group of the patients were distributed between 61 and 70 years (n = 38; 40.86%) followed by 51 and 60 years (n = 28; 30.11%). The distribution of patients in each age-decade is presented in Figure 1.

Sex

There were 47 (50.54%) male and 46 (49.46%) female patients. The overall distribution of synchysis scintillans was slightly greater in females (0.0032%; 46/1,423,295) as compared to males (0.0028%; 47/1,659,432) but was not statistically significant (p = 0.524209). Among the patients diagnosed with synchysis scintillans, the mean and median age were 61.14±14.87 and 65 (IQR: 56 to 72) years for men and 60.13±7.59 and 60 (IQR: 55 to 66) years for women, respectively. The overall mode was 66 years and 65 years in men and 61 years in women.

Urban-rural distribution

Of the 93 patients with synchysis scintillans, 42 (45.16%) were from an urban locality, 34 (36.56%) were from a rural locality and 17 (18.28%) patients presented from the metropolitan region. The overall prevalence of synchysis scintillans in the metropolitan community (0.005%; 17/358,434) was higher as compared to the urban (0.003%; 42/1,341,267) or rural community (0.002%; 34/1,383,026) and was statistically significant (p = 0.045359).

Socioeconomic status

Of the 93 patients with synchysis scintillans, there were 18 (19.35%) patients from the lower socioeconomic class, 68 (73.12%) patients from the lower middle class, seven (7.53%) patients from the upper middle class, and 0 (0%) patients from the upper class. The overall prevalence of synchysis scintillans was slightly higher in the higher socioeconomic strata (0.0032%; 75/2,363,156) as compared to lower socioeconomic strata (0.0025%; 18/719,571) but was not statistically significant (p = 0.36336).

Occupation

Of the 93 patients with synchysis scintillans, 36 (38.71%) were homemakers; 18 (19.35%) were retired individuals; 15 (16.13%) were professionals; 10 (10.75%) were agricultural workers; seven (7.53%) were manual laborers; one (1.08%) was student, and in the remaining six (6.45%) patients, the occupational category was not available/applicable. The overall prevalence of synchysis scintillans in retired individuals (0.018%, 18/99,637) was significantly higher (p<0.00001) in comparison to other professions.

Laterality

Of the 93 patients with synchysis scintillans, 40 (43.01%) were affected in the left eye and 36 (38.71%) were affected in the right eye. In 17 (18.28%) patients, the affliction was bilateral in nature.

Systemic disease

Among the 93 patients, diabetes mellitus was documented in 13 (13.98%) patients, hypertension in 15 (16.13%) patients, arthritis, coronary artery disease, and hypothyroidism in one (1.08%) patient each.

Clinical history

A past history of ocular trauma was documented in three (3.23%) patients. Synchysis scintillans was documented in the anterior chamber in one (1.08%) patient.

Presenting visual acuity

Of the 110 eyes, 74 (67.27%) eyes had mild or no visual impairment (<20/70), 13 (11.82%) eyes had moderate visual impairment (>20/70 to 20/200), four (3.64%) eyes had severe visual impairment (>20/200 to 20/400), 12 (10.91%) eyes had blindness (>20/400 to 20/1200), one (0.91%) eye had blindness (>20/1200 to light perception (PL)), two (1.82%) eyes had blindness (no light perception (NPL)), and in four (3.64%) eyes’ the visual acuity was undetermined or unspecified.

Ocular comorbidities

Among the 110 eyes, an associated cataract was documented in 61 (55.45%) eyes, glaucoma in eight (7.27%) eyes, diabetic retinopathy in four (3.64%) eyes, retinal detachment in two (1.82%) eyes, vitreous hemorrhage in one (0.91%) eye, and phthisis bulbi in one (0.91%) eye.

Surgical treatment

Among the 110 eyes, surgical intervention was performed for associated ocular comorbidities in 18 (16.36%) eyes. Cataract surgery was performed in nine (8.18%) eyes, vitreoretinal surgery in two (1.82%) eyes, and trabeculectomy in two (1.82%) eyes.

## Discussion

This study sought to describe the clinical profile and demographic distribution of synchysis scintillans in a large cohort of patients presenting to a multitier hospital network in India using electronic medical records-driven big data analytics. The primary purpose of the study was to determine the relative proportion and demographic profile of the synchysis scintillans in the clinical care setup. The overall prevalence of synchysis scintillans was 0.003% in patients who presented between 2010 and 2021 (an 11-year period). The disease is predominantly unilateral and equally affects males and females. It causes mild or no visual impairment in the majority of the affected eyes and cataract is associated in half of them.

There is inadequate literature related to synchysis scintillans and is mostly limited to case reports. Wand et al. published their experience in over 59,563 eye admissions and 10,780 specimens and concluded that synchysis scintillans was not diagnosed in any of the cohorts and was found in 12 pathology specimens as cholesterol crystals in the vitreous cavity [1]. They also concluded that the disease is more commonly bilateral and occurs in eyes with a past history of injury, vitreous hemorrhage, or retinal detachment. In our cohort, we have found that synchysis scintillans is predominantly a unilateral condition (81.72%) and also presents more commonly in the sixth (30.11%) and seventh decade (40.86%) of life. We also found no significant past history of trauma in the patients (3.23%) and a minor proportion of eyes had retinal detachment (1.82%) and vitreous hemorrhage (0.91%). Contrary to the conclusion by Wand et al. that synchysis scintillans occurs only in severely damaged, blind eyes, our study shows that it can occur in normal eyes as well as an age-related degeneration of the vitreous.

The presence of synchysis scintillans in the anterior chamber has been described in the literature [10-13]. In our cohort, we found only one patient (1.08%) with synchysis scintillans reported in the anterior chamber, and the remaining were diagnosed as present in the vitreous cavity. Potter questioned the existence of the condition and opined that though it is traditionally compared to asteroid hyalosis, synchysis scintillans is extremely rare and is observed only in severely damaged and blind eyes [14]. Our prevalence of 0.003% confirms the rarity of the condition as well but it is found in eyes without any prior history of injury or other risk factors. We found that cataract was the most commonly associated with ocular comorbidity in our cohort and was seen in more than half of the cases (55.45%).

Bergandi et al. studied oxidative stress, lipid peroxidation, and loss of hyaluronic acid in the human vitreous affected by synchysis scintillans. They have reported that the antioxidative activities of superoxide dismutase (SOD) and catalase are significantly lower and the amount of hyaluronic acid also was lower in the vitreous affected by synchysis scintillans [5]. They proposed that the cholesterol crystals could trigger inflammasome-mediated innate responses leading to retinal damage. The mean age of the patients with synchysis scintillans in their study was 75.9±2.1 years indicating an elderly cohort. Our cohort also significantly showed that the condition occurred between the sixth and seventh decades of life with a mean age of 60.64±11.85 years indicating that this might actually be a degenerative process in the vitreous in normal eyes contrary to the belief of it being more commonly found in severely damaged or inflamed eyes.

This study lends insight into the socio-demographic and clinical presentation of patients with synchysis scintillans in a large cohort of patients in India. The study does have a few limitations due to its hospital-based method of selection of subjects, which may have introduced a certain level of ascertainment bias but the greatest strength are the complete utilization of the digital data entry in a structured manner by trained ophthalmologists and automated extraction methods for analysis. This cohort of patients lends new insight to the pattern of this disease that can help guide future questions that can be asked related to the pathophysiology, its association with various other vitreoretinal disorders, and its effect on the retina.

## Conclusions

In conclusion, this study aimed to describe the epidemiology and clinical presentation of synchysis scintillans in 3 million new patients presenting to a multitier ophthalmology hospital network in India. The findings show that synchysis scintillans equally affect males and females presenting during the seventh decade of life and is predominantly unilateral. The majority of the eyes have mild or no visual impairment and over half of the eyes have an associated cataract.
